# Localized *de novo* phospholipid synthesis drives autophagosome biogenesis

**DOI:** 10.1080/15548627.2020.1725379

**Published:** 2020-02-06

**Authors:** Maximilian Schütter, Martin Graef

**Affiliations:** a Group of Autophagy and Cellular Ageing, Max Planck Institute for Biology of Ageing, Cologne, Germany; b Cologne Excellence Cluster on Cellular Stress Responses in Aging-Associated Diseases (CECAD), University of Cologne, Cologne, Germany

**Keywords:** Acyl-CoA synthetase, autophagosome biogenesis, autophagy, fatty acid channeling, fatty acid synthase, phospholipid synthesis

## Abstract

During (macro)autophagy, cells form transient organelles, termed autophagosomes, to target a broad spectrum of substrates for degradation critical to cellular and organismal health. Driven by rapid membrane assembly, an initially small vesicle (phagophore) elongates into a large cup-shaped structure to engulf substrates within a few minutes in a double-membrane autophagosome. In particular, how autophagic membranes expand has been a longstanding question. Here, we summarize our recent work that delineates a pathway that drives phagophore expansion by localized *de novo* phospholipid synthesis. Specifically, we found that the conserved acyl-CoA synthetase Faa1 localizes to nucleated phagophores to locally activate fatty acids for *de novo* phospholipid synthesis in the neighboring ER. These newly synthesized phospholipids are then preferentially incorporated into autophagic membranes and drive the expansion of the phagophore into a functional autophagosome. In summary, our work uncovers molecular principles of how cells coordinate phospholipid synthesis and flux with autophagic membrane formation during autophagy.

**Abbreviations:** ACS: acyl-CoA synthestases; CoA: coenzyme A; ER: endoplasmic reticulum

The identification of the membrane sources for the formation of the double-membrane autophagosome has been a longstanding quest in the autophagy field. A number of organelles including the ER, Golgi, endosomes, and mitochondria have been implicated in contributing mainly preformed membranes. However, it has been a major challenge to clearly define at which stage and to which extent these membrane sources may function in autophagy. In particular, the mechanisms underlying the rapid membrane assembly during the expansion of the phagophore, a stage during which the majority of the autophagic membrane has to be generated, are elusive. In our recently published work [1], we investigated the role of acyl-CoA synthetases (ACS) and *de novo* phospholipid synthesis for autophagosome biogenesis. ACS constitute a conserved protein family that catalyzes the activation of free fatty acids by thioesterification with coenzyme A (CoA) to generate activated fatty acids (acyl-CoA). It is thought that, by means of dynamic and differential localization within cells, ACS form a multi-protein network that regulates the flux of fatty acids into diverse cellular pathways including lipid synthesis, vesicular fusion, or membrane editing.

Analyzing the ACS network of budding yeast by live cell imaging, we found that two ACS, Faa1 and Faa4, accumulate on forming autophagosomes, showing that a subset of ACS localizes to autophagic membranes. To test for a function during autophagy, we generated strains expressing either wildtype *FAA1* (*FAA1* cells) or a *FAA1* variant tethered to the plasma membrane (*PM-FAA1* cells) lacking other redundant ACS. In addition, we inhibited fatty acid synthase (FAS) with cerulenin, because FAS constitutes a yeast-specific parallel pathway for acyl-CoA production. Strikingly, *FAA1* cells display wild-type-like autophagy flux upon starvation, whereas *PM-FAA1* cells are strongly impaired, indicating that the local activity of Faa1 on autophagic membranes is essential for autophagy. Indeed, systematic tethering experiments revealed a requirement for local fatty acid activation by Faa1 at, or in close proximity to, autophagic membranes for autophagy. Importantly, both recruitment to autophagic membranes and autophagy flux are fully restored upon heterologous expression of the peripheral isoform, v1, of human ACSL4, indicating conservation of ACS function for autophagy from yeast to human.

Next, we aimed at identifying the mechanistic functions of local fatty acid activation for autophagy. Importantly, time-lapse imaging revealed impaired autophagosome biogenesis in the absence of Faa1-mediated fatty acid channeling. In particular, multiple lines of experiments including a direct measurement of phagophore dynamics established that, downstream of the autophagy core machinery and phagophore nucleation, the local activity of Faa1 specifically drives and is limiting for the expansion of the phagophore membrane. To test whether Faa1-dependent changes in phagophore expansion may be linked to an altered membrane composition of autophagosomes, we established a protocol to purify autophagic membranes via Atg8 immuno-isolation coupled with high resolution lipidomics. Our approach provides the first insights into the composition of autophagic membranes, which are characterized by a high content of desaturated phospholipids conducive to highly dynamic phagophore behavior. However, the phospholipid composition of autophagic membranes is unaffected by local Faa1 activity. However, when we traced isotopic fatty acids in whole cell and isolated autophagic membranes, we found that Faa1-mediated fatty acid channeling promotes the generation and preferential incorporation of newly synthesized phospholipids into autophagic membranes. Thus, these observations indicated that Faa1 channels activated fatty acids into the synthesis of phospholipids to drive the expansion of phagophore membranes.

The ER is the central organelle for *de novo* phospholipid synthesis. To critically test our model, we conditionally inhibited the first two committed steps of *de novo* phospholipid synthesis within the ER. Strikingly, impaired *de novo* phospholipid synthesis strongly reduces autophagy flux and the dynamics of phagophore expansion, demonstrating its essential nature for autophagy. Taken together, our work strongly supports a model in which phagophores are nucleated from preformed membranes, consistent with substantial prior published work, which appear to be insufficient, however, to sustain efficient phagophore expansion. Instead, the conserved ACS Faa1 is recruited to nucleated phagophores to channel fatty acids into *de novo* phospholipid synthesis within the ER. These newly synthesized phospholipids are an essential source for the efficient expansion of the phagophore and the formation of functional autophagosomes. Thus, our model provides a simple mechanism to couple “on demand” phospholipid synthesis to autophagosome biogenesis in a spatiotemporal manner. It remains to be demonstrated how a net transfer of phospholipids from the ER to the neighboring phagophore is achieved *in vivo*, but it likely involves the conserved ER-phagophore tether Atg2-Atg18 and/or direct ER-phagophore membrane connections. Thus, we propose that detailed insights into the mechanisms that control phospholipid dynamics at and across ER-phagophore contact sites will be key for a further understanding of how autophagosomes form.
